# The Effect of Chronic Inflammation and Oxidative and Endoplasmic Reticulum Stress in the Course of Metabolic Syndrome and Its Therapy

**DOI:** 10.1155/2018/4274361

**Published:** 2018-10-22

**Authors:** Michalina Alicka, Krzysztof Marycz

**Affiliations:** ^1^Department of Experimental Biology, The Faculty of Biology and Animal Science, University of Environmental and Life Sciences Wroclaw, Wroclaw, Poland; ^2^Faculty of Veterinary Medicine, Equine Clinic-Equine Surgery, Justus-Liebig-University, 35392 Gießen, Germany

## Abstract

Metabolic syndrome (MetS) is highly associated with a modern lifestyle. The prevalence of MetS has reached epidemic proportion and is still rising. The main cause of MetS and finally type 2 diabetes occurrence is excessive nutrient intake, lack of physical activity, and inflammatory cytokines secretion. These factors lead to redistribution of body fat and oxidative and endoplasmic reticulum (ER) stress occurrence, resulting in insulin resistance, increase adipocyte differentiation, and much elevated levels of proinflammatory cytokines. Cellular therapies, especially mesenchymal stem cell (MSC) transplantation, seem to be promising in the MetS and type 2 diabetes treatments, due to their immunomodulatory effect and multipotent capacity; adipose-derived stem cells (ASCs) play a crucial role in MSC-based cellular therapies. In this review, we focused on etiopathology of MetS, especially on the crosstalk between chronic inflammation, oxidative stress, and ER stress and their effect on MetS-related disease occurrence, as well as future perspectives of cellular therapies. We also provide an overview of therapeutic approaches that target endoplasmic reticulum and oxidative stress.

## 1. Introduction

Metabolic syndrome—also called insulin resistance syndrome or syndrome X—was described for the first time in 1988 [1]. The term describes all of the metabolic disorders that are associated with visceral adiposity. The metabolic syndrome frequency depends on sociodemographic and geographic factors. The conditions that have been described as metabolic risk factors of MetS development involve insulin resistance, hypertension, dyslipidemia (hypertriglyceridemia, low high-density lipoprotein cholesterol), abdominal obesity, elevated glucose levels, high blood pressure, and proinflammatory and prothrombotic state [2]. Characteristics of insulin-resistant phenotype include impaired glucose metabolism or tolerance, elevated fasting glucose levels and/or hyperglycemia, and decreased insulin-mediated glucose reductions in the suppression of glucose production inside the body [3]. Genetics, postmenopausal status [4], excessive alcohol consumption [5], prolonged chronic stress [6], and physical inactivity [7] may have a causal effect, but these depend on the ethnic group [8].

Nutrition Examination Survey and National Health data estimate that the highest MetS prevalence has Mexican-American women. The data estimates that 34% of adults in the United States had a diagnosis of MetS [9]. International Diabetes Federation extrapolates that MetS prevalence in European population is 41% in men and 38% in women [10]. MetS is associated with a high risk of developing several lifestyle diseases including type 2 diabetes and cerebrovascular disease. Moreover, individuals with the MetS carry an approximately twofold increase in the risk of cardiovascular disease [2]. Globally, 8.3% of adults (382 mln) live with type 2 diabetes but the number is still rising. It is estimated that the number of diabetes patients will increase to 592 mln in 20 years (IDF). Furthermore, it is well known that type 2 diabetes is currently the leading cause of death among people under 60 years of age. The emerging problem has become the reason for new drug development, such as dipeptidyl-peptidase-4 (DPP-4) inhibitors, sodium glucose transporter-2 (SGLT2) inhibitors, glucagon-like peptide (GLP-1) mimesis, and cellular therapy (MSC) application [11]. Although it is obvious that the accumulation of visceral and ectopic fat is an important contributing factor to metabolic and cardiovascular risk implementation of fat distribution, assessment into clinical practice is still a challenge. Moreover, the prevalence of obesity has increased over the last 20 years [12]. The main recommendation for metabolic syndrome prevention and treatment is changes in the lifestyle. The changes include regular physical activity [13, 14] and calorie restriction [15]. Herein, we demonstrated the effect of chronic inflammation, oxidative stress, and prolonged endoplasmic reticulum stress on MetS development, as well as the potential of cellular therapy on MetS-associated diseases, especially type 2 diabetes treatment.

## 2. Adipose Tissue and Metabolic Syndrome: Chronic Inflammation

Adipose tissue is an active metabolic and endocrine organ responsible for the cross-talk between various systems, including the immune and the cardiovascular systems [16]. Adipocytes play an important role in the control of energy balance and lipid/glucose homeostasis. Adipose tissue excess or obesity has an effect of pathology in caloric balance in susceptible individuals that contribute to metabolic syndrome [17]. The adipocyte is the only cell whose size evolves drastically. Studies have reported that the degree of inflammation in the adipose tissue depends on the size of the adipocytes [18]. Prolonged obesity causes cell hypertrophy (cell size increase), which might lead to MetS occurrence. Because of the alternations, new adipocytes are required to work against the hypertrophic adipocytes [19, 20]. Probably, the adipose tissue depots of overweight individuals have already committed the resource of their stem cells to the adipocyte lineage and, consequently, have no capacity to create new adipose cells [21]. Adipocyte hypertrophy leads to local adipose tissue hypoxia, caused by relative deficiency of vasculature [22, 23]. Furthermore, hypoxia can induce cell necrosis with the production of adipokines [24].

Adipose tissue is a type of connective tissue which consists predominantly of mature adipocytes and stromal vascular fraction (SVF) cells, such as preadipocytes, fibroblasts, blood cells, macrophages, and endothelial cells [25]. Furthermore, the adipocyte tissue is a source of multipotent adipose tissue-derived mesenchymal stem cells (ASCs). ASCs were first described by Zuk and colleagues at the beginning of the 21^st^ century [26] as multipotent, self-renewing, and undifferentiated progenitor cells that are phenotypically and morphologically similar to bone marrow-derived mesenchymal stem cells (BMSCs). Moreover, the cells have an ability to form single colonies (CFU-Fs) and adhere to the plastic surface. The Internal Fat Applied Technology Society has suggested that both ASCs and BMSCs display the stem cell-specific types of surface markers, including CD90, CD105, CD73, CD44, and CD166, and lack of the typical hematopoietic markers CD45 and CD34. Moreover, ASCs have an ability to differentiate into cells of mesoderm, including adipocytes, osteocytes, chondrocytes, and myocytes, that exhibit their potential for cell-based therapies [27, 28]. Both populations of MSCs, because of their immunomodulatory activity and the potential to differentiate into insulin-producing cells, might become a potential therapy strategy in the course of type 2 diabetes and MetS [29–31]. Bone marrow-derived MSCs are suboptimal for clinical use in regenerative medicine because of a highly invasive aspiration procedure [32]. ASC isolation from subcutaneous adipose tissue is a minimally invasive method [33, 34]. The group [26] has demonstrated that the adipose tissue is an alternative source of MSC.

Abdominal obesity is associated with an increase in both adipocyte size and adipocyte number; furthermore, MSCs are able to differentiate into adipocytes. Thus, new adipocytes arise from ASC pools regardless of age [34]. Furthermore, Liechty and colleagues have demonstrated that xenotransplantation of MSCs from human into fetal sheep marrow was successfully performed. Moreover, the cells were able to differentiate into adipocytes in adipose tissue, confirming that MSCs are an important cell source for adipogenesis [35, 36]. As mentioned above, proliferations and differentiation capacity decrease with the age because of both an accumulation of multiple oxidative stress factors and decrease antioxidative protection. The events lead to the decrease of proliferation activity, decreased differentiation capacity, and higher sensitivity to apoptosis. The alternations very limit their therapeutic potential [11, 37–39].

MetS progression is associated with decreased CD34, as well as endothelial progenitor cell level. Furthermore, some factors, like elevated proinflammatory cytokine, including IL-6 and IFN*α* induce MSC proliferation, although normally they are in an undifferentiated and inactive state [40]. Thus, both increased caloric uptake and inflammation can cause a constant providing of alarm factors and MSC activity. Furthermore, reduction of MSC pool can lead to irreparable impairment of body regeneration [41]. A equine model has shown that MSCs isolated from EMS (equine metabolic syndrome) horses exhibited decreased clonogenic potential, increased population doubling time (PDT), and depletion of MSC-specific surface markers, such as CD90, CD105, and CD73 [42–45]. Moreover, murine studies have demonstrated decreased CD105 expression consistent with decreased potential for chondrogenic differentiation in obese mice [46]. It is interesting that MetS cells were characterized by overexpression of immune cell receptor CD44, a cell surface marker which plays an important role in inflammatory cell activation. Interestingly, other independent studies have confirmed the theory of overexpression CD44 in an obese mouse. Moreover, knockout CD44 mouse did not develop neither obesity nor type 2 diabetes despite high-fat diet feeding [47–49]. Another research has revealed that overexpression of CD44 is significantly correlated with local inflammation and systemic insulin resistance in human adipose tissue [49] (Table 1).

Multiple scientists described the function of the immune system in metabolic syndrome. It is not still clear what causes MetS, but it is known that a chronic inflammatory state is associated with metabolic syndrome occurrences. The report is based on evidence of increased various proinflammatory cytokines (e.g., interleukin 1*β* (IL-1*β*), IL-8, monocyte chemoattractant protein-1 (MCP-1), tumor necrosis factor *α* (TNF*α*), and prothrombotic mediator plasminogen activator inhibitor-1 (PAI-1)), as well as biomarkers of inflammation (e.g. C-reactive protein (CRP)) concentration in MetS individuals [50, 51]. There are three major sites that have been involved in initiating of chronic inflammation in MetS: liver, intestine, and adipose tissue [52–55]. Moreover, the secretion of inflammatory mediators from one tissue promotes inflammation in other sites, thereby increasing the chronic inflammation in the body causing tissue dysfunction/injury [54].

Recently, many studies reported that adipocyte hypertrophy and hyperplasia in response to nutritional excess result in abnormal adipose cell function and cause insulin resistance [25, 56, 57]. Hypertrophic adipocytes secrete proinflammatory cytokines, such as TNF*α*, IL-6, IL-8, and MCP-1. High levels of proinflammatory cytokines cause serine phosphorylation of insulin receptor substrate-1 via nuclear factor *κ*B and Jun N-terminal kinase signaling. The reaction leads to the development of insulin resistance [58, 59] (Table 2). Hypoxia can contribute to the induction of cell necrosis with the production of TNF*α*, IL-6, PAI-6, and macrophage infiltration [22].

TNF*α* acts as a paracrine mediator to enhance insulin resistance in adipose cells. Furthermore, it is an important mediator of several cardiovascular events, such as heart failure and atherosclerosis [60]. TNF*α* is also key in the pathophysiology of inflammatory dermatoses associated with MetS, including hidradenitis suppurativa and psoriasis [61].

IL-6 is a potent cytokine that plays a significant role in the pathogenesis of insulin resistance and type 2 diabetes. An excess of IL-6 has been measured in adipose tissue in patients with obesity and type 2 diabetes, as well as in patients with MetS [62]. Moreover, a murine model has shown that elevated IL-6 level is associated with several diseases which include cardiovascular events, atherosclerosis, and hypertension and chronic IL-6 exposure causes hyperglycemia associated with insulin resistance [63, 64].

PAI-1 is a serine protease inhibitor that exhibits inhibitory activity toward the plasminogen activator urokinase. Elevated circulating PAI-1 has been reported in both obese MetS patients and patients with type 2 diabetes. Additionally, there is a positive correlation between intension of the plasma concentration of PAI-1 and MetS. The mechanism of the inhibitor overexpression in MetS involves several mediators, but the exact way is still unknown. Recent studies have suggested that PAI-1 is committed in control of insulin signaling and adipose tissue development [65].

MCP-1 is chemokine that allures macrophages into the adipose tissue in obesity. A murine model has exhibited that both MCP-1-deficient and MCP-1 receptor CCR2-deficient mice are protected against inflammation and macrophage accumulation in adipose tissue and display resistance to DIO-caused insulin resistance [66, 67]. MCP-1 secretion depends on the size of adipocytes; large adipocytes exhibit elevated secretion, which means that MCP-1 expression and secretion are upregulated in obesity and reduction after weight loss [18].

## 3. Impact of Oxidative Stress in the Course of MetS

The detailed mechanism of adipocyte dysregulation occurrences is not clear, but scientists postulated that both inflammatory mediators and obesity-induced oxidative stress affect MetS development. Oxidative stress (OS) is defined as an imbalance between antioxidant and prooxidant factors. The imbalance can lead to oxidative damage to proteins, nucleic acids, and lipids in different biological systems, therefore causing structural and functional impairment of many molecules [71, 72]. OS plays an important role in MetS and MetS-related diseases. Elevated oxidative damage, such depressed *α*-tocopherol and vitamin C concentration, decreased antioxidant protection and superoxide dismutase (SOD) activity, and increased malondialdehyde levels, lipid peroxidation, protein carbonyls, and xanthine oxidase activity are strongly correlated with MetS occurrence [73, 74].

Several animal and human studies have shown a positive correlation between adipose tissue accumulations an oxidative stress which has resulted in increased production of reactive oxygen species (ROS) and overexpression of NADPH oxidase with simultaneous decreased expression of antioxidant enzymes. Moreover, *in vitro* research has revealed that the application of higher concentration of fatty acids in adipocytes culture leads to the increase in oxidative stress via the NADPH pathway. An animal model has shown that feeding mice with a high-glucose diet which suffered from hyperglycemia resulted in free radicals production and, consequently, oxidative stress occurrence [75]. Furthermore, overweight mice treated with NADPH oxidase inhibitor have decreased ROS production with a reduction of diabetes symptoms [76, 77]. There is a close correlation between oxidative stress in the metabolic syndrome patients and the progress of complications, like vascular endothelial activation that can cause atherosclerosis [78]. Moreover, type 2 diabetes mellitus patients have elevated lipid peroxidation in comparison to age-matched control subject and reduced plasma glutathione (GSH) level, as well as GSH-metabolizing enzymes [79, 80]. Furthermore, several studies have shown that mitochondrial dysfunction is highly associated with obesity, strictly linked to increased ROS production and the progression of insulin resistance [81]. Similar to the murine model, a number of studies have provided that treatment reducing reactive oxygen species production increases insulin sensitivity and decreases hyperlipidemia and hepatic steatosis [76, 82, 83]. Oxidative stress is also highly associated with endoplasmic reticulum stress occurrence.

## 4. Endoplasmatic Reticulum Stress in the Course of MetS

**Figure 1:**
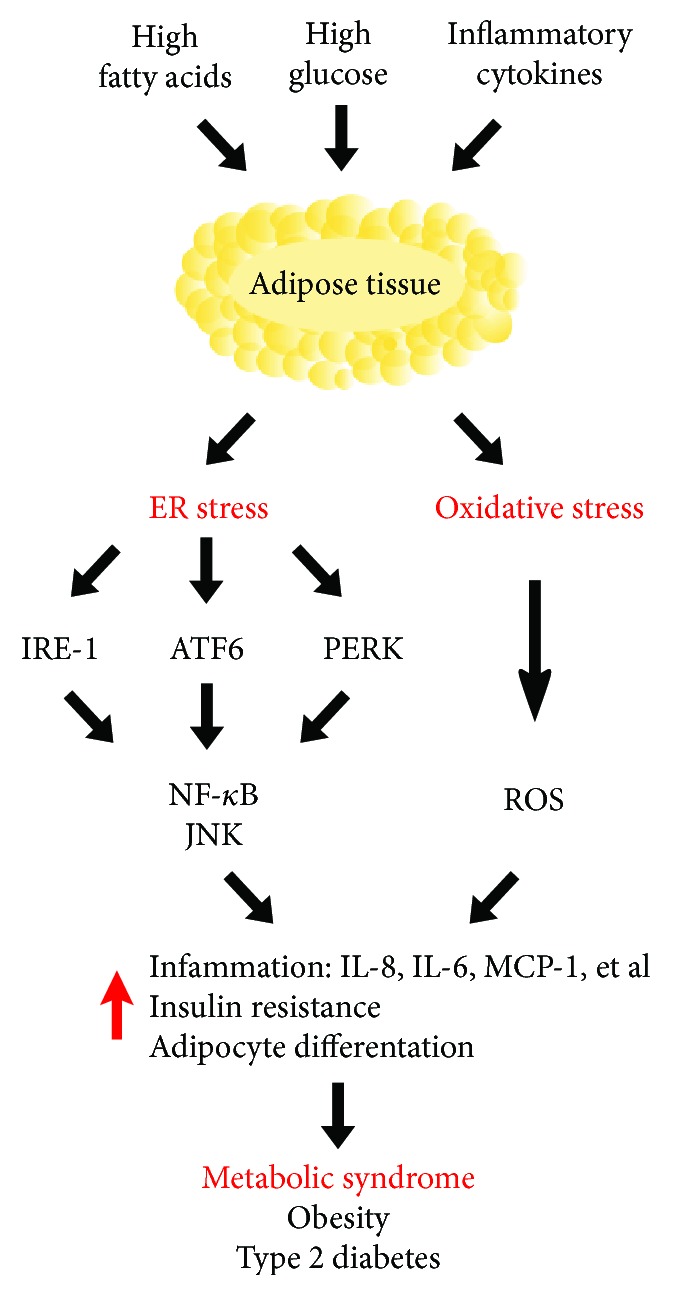
Schema of the correlation between excessive intake of fatty acids and glucose and inflammatory cytokine secretion on metabolic syndrome occurrence. Overconsumption of fat and sugar and proinflammatory cytokine production play a crucial role in ER and oxidative stress occurrence. Elevated NF-*κ*Β, JNK, and ROS concentration leads to increase inflammation, insulin resistance, and adipocyte differentiation, which cause MetS and finally obesity and type 2 diabetes.

The endoplasmic reticulum (ER) is an intracellular organelle responsible for lipid and protein biosynthesis, protein folding, maturation, and quality control. Moreover, it is a critical site for calcium cation (Ca^2+^) homeostasis. The Ca^2+^ are maintained at relatively high concentrations inside the lumen and can be released out of the ER during cell signaling responses [84]. There are two conceptual types of ER membranes: smooth and rough. The smooth ER is a key site for the biosynthesis of lipids, while the rough ER is studded with ribosomes which are responsible for protein synthesis. In addition, the protein folds and maturates in the ER lumen with the assistance of ER luminal chaperone proteins [85]. The accumulation of unfolded and incompletely folded proteins changes to redox status and luminal Ca^2+^ concentration (ER stress) and activates intracellular signaling pathways to restore homeostasis. In the ER, the unfolded protein response (UPR) acts to increase the capacity of ER protein folding and posttranslational modification [86, 87]. The UPR has three signaling arms: the inositol-requiring enzyme 1*α* (IRE1*α*), the protein kinase RNA-like endoplasmic reticulum kinase (PERK), and the activating transcription factor 6 (ATF6). Studies in the last decade indicate that impairment of sensing and signaling pathway downstream of ER stress has a significant impact on the pathogenesis of numerous human metabolic disorders, including insulin resistance (IR), obesity, and diabetes [87, 88] (Figure 1).

### 4.1. IRE1*α* Signaling Pathway

IRE1*α* is the most evolutionarily conserved UPR branch and exhibits endoribonuclease activity. There are two *IRE1* genes (*IRE1α* and *IRE1β*), but only *IRE1α* is expressed ubiquitously. Under physiologic conditions, it remains in an inactive form, because of an interaction with immunoglobulin heavy chain-binding protein (BiP). Upon activation (accumulation of unfolded and misfolded proteins), BiP dissociates from IRE1*α*, leading to dimerization, transautophosphorylation of the luminal domain of IRE1*α*, and activation of the RNase and kinase activities. The protein kinase and RNase domains are localized within their cytosolic region and splice X-box binding protein 1 (XBP-1) transcripts. Thus, IRE1*α* leads to the generation of the transcription factor XBP-1 and indirectly causes overexpression of ER luminal chaperones, as well as ER-associated degradation (ERAD) machinery elements [89]. Moreover, XBP-1 increases biogenesis capacity of ER and Golgi apparatus, thereby increasing the pace of protein secretion [90]. Recent studies indicate that *IRE1* is able to microRNA (miR) degradation causing activation of inflammatory and apoptotic pathways [91]. Interestingly, as a protein kinase, *IRE1*contributes the ER protein folding, thereby leading to inflammation through interaction with TNF*α* receptor-associated factor 2 (TRAF2). The process activates the nuclear factor *κ*Β (NF*κ*Β) and c-Jun N-terminal kinase (JNK) pathways through apoptosis signal-regulating kinase-1 (ASK1) [92–94].

### 4.2. PERK Branch of the ER Stress Response

PERK is a serine threonine kinase that phosphorylates downstream targets such as IRE1. Interestingly, PERK signaling pathway activation occurs with a slower kinetic than IRE1*α* and ATF6 [95, 96]. It has a luminal ER stress-sensing domain activated through transautophosphorylation and, upon activation, phosphorylates eukaryotic translation initiation factor 2 alpha (eIF2*α*). The process causes reduction of global protein biosynthesis and ER protein folding load [97]. Activated eIF2*α* enhances the translation of activating transcription factor-4 (ATF4) which leads to induction of the UPR effector C/EBP*α*-homologous protein (CHOP). Interestingly, in pathological states, prolonged CHOP expression causes apoptosis occurrence through several mechanisms, such as decreased expression of the antiapoptotic factor B cell lymphoma-2 (Bcl-2) [88].

### 4.3. ATF6 Signaling Arm

Unlike both the IRE1*α* and PERK, ATF6 is released from BiP/Grp78 binding and translocates to Golgi complex after ER stress. In the Golgi apparatus, ATF6 is cleaved by site 1 and site 2 proteases (S1P and S2P) at the transmembrane site to generate a transcriptionally active polypeptide. The released ATF6 cytosolic fragment p50 can translocate to the nucleus to increase the expression of numerous ER chaperone genes, such as *BiP*, ERAD components, and *XBP-1* mRNA [86, 92, 97].

ER stress is highly correlated with metabolic disorders including type 2 diabetes and obesity. Recent studies have revealed that ER stress-mediated activation of JNK has been associated with insulin resistance through phosphorylation of insulin receptor substrate-1 (IRS1) on Ser307, which leads to the reduction of tyrosine phosphorylation and IRS1 activation. In addition, ER stress factors, such as XBP-1s, phosphor-JNK, and phosphor-eIF2*α*, exhibit upregulation in the adipose tissue and liver of obese insulin-resistant nondiabetic individuals. Moreover, the factor levels significantly decreased after weight loss [98, 99]. In addition, ER stress-induced insulin resistance in the muscle requires the induction of the mTORC1 pathway [100].

The number of evidence that ER stress is associated with the pathogenesis of metabolic syndrome has been increased in the last few years, and several drugs, especially UPR regulators, have been tested [101]. BiP is a major regulator of the UPR, and the regulation of BiP expression plays an important role in ER stress modulation [102]. For example, valproate, a small molecule BiP activator, protects beta cells from palmitate-induced ER stress apoptosis. BiP is highly expressed in the ER and can be used as the endoplasmic reticulum marker [102]. Moreover, small molecule inhibitors of PERK and eIF2*α*, such as GSK2606414 and GSK2656157 (preclinical candidate), have been developed. Both of them have been commonly used to reduce ER stress by inhibition of receptor-interacting serine/threonine-protein kinase 1 (RIPK1) and thereby apoptosis [102, 103]. Guanabenz has been discovered as a molecule that targets eIF2*α* phosphorylation and leads to the reduction of protein production and thus reduces ER stress. Furthermore, salubrinal protects cells from ER stress-induced apoptosis by inhibition of dephosphorylation of eIF2*α* [104]. Integrated stress response inhibitor (ISRIB) has been described as an eIF2B activator and UPR inhibitor in the PERK branch [105]. Moreover, specific inhibitors of CHOP have also been developed. CHOP is activated through phosphorylation mediated by p38 mitogen-activated protein kinase (MAPK) and its inhibition may be beneficial to diabetes patients because of reduction of ER stress-mediated apoptosis [106, 107]. Two chemical compounds with similar structures named STF-083010 and 4*μ*8C selectively inhibit IRE1's RNase capacity and thus inhibits UPR [108]. Furthermore, both drugs are safe for human that make them promising candidates for clinical treatment. Other studies have shown that both compounds have been used in cells to protect them from apoptosis and ER stress [109]. Each ER stress signaling pathway carries out specialized function in metabolic diseases, and new drugs specific to the ER stress branches have been described as promising candidates in ER stress therapy and are currently being developed [101].

ER stress can lead to mitochondrial damage and oxidative stress induction. IRE1*α* interacts with Bak and Bax (proapoptotic Bcl-2 family members) and enhances mitochondrial-dependent cellular death. In addition, during ER stress, calcium cations released from the ER lumen can be taken up by the nearby mitochondria, which causes mitochondrial damage and thereby increases the production of ROS and proapoptotic signaling. Moreover, both mitochondria and ER are physically and functionally linked by mitochondria-associated ER membranes (MAMs) [110]. Recent studies have pointed out that ER protein folding process is highly associated to ROS production. Redox homeostasis is essential in the protein folding pathway as well as disulfide bone formation [111–113]. Some alternations in that process lead to ROS imbalance and increase ROS production. Disulfide bone formation, crucial for the production of mature and functional proteins, is a reversible process catalyzed by several ER oxidoreductases (e.g., ER protein (ERP) 57 and ERP72) [114]. The redox state is modulated by numerous redox mechanisms. The GSH/GSSG cycle is one of the redox mechanisms that plays a crucial role in the protein folding process. GSH can undergo oxidation to GSSG maintaining redox homeostasis. The balance between GSH and GSSG (1 : 1 in the RE lumen and 1 : 50 in the cytoplasm, respectively) is essential in maintaining ER redox homeostasis [113]. If production of misfolded proteins occurs, GSH can reduce nonnative bonds allowing them to refold again. The protein refolding process is very slow and needs electron acceptors. When large numbers of misfolded proteins are accumulated in the ER, GSH mechanism is compromised; ROS production and ER stress occur [112, 115]. Beyond the GSH/GSSG mechanism, protein disulfide-isomerase (PDI) can cause ER stress and OS occurrence. PDI catalyzes the formation of disulfide bone as a multifunctional oxidoreductase chaperone protein. During the oxidative protein folding process, two electrons are transferred to the oxygen molecules, thereby producing hydrogen peroxide (a type of ROS) that leads to OS [116]. The process leads to redox balance alternation and thus ER stress [112, 117]. Both of the two mechanisms can provide an important indicator of the oxidative stress in ER.

Interestingly, the interdependence of ER stress and oxidative stress often causes the activation of inflammation [118]. Inflammation can activate UPR by all three branches (PERK, IRE1*α*, and AT6 signaling). In turn, UPR can modulate crucial proinflammatory pathways, such as the JNK/activator protein 1 (AP1) and NF*κΒ* [119]. The NF*κ*Β pathway can be triggered through PERK, IRE1*α*, and AT6, whereas the JNK/AP1 is mainly induced by IRE1 [101]. Recent studies have pointed out that a high-fat diet can induce inflammatory response in obese rats and mice by ER stress and downstream of the Toll-like receptor 4 (TLR4) signaling pathway [120, 121]. Levels of some interleukins, like IL-23, IL-24, and IL-33, are increased in diabetic beta cells and can lead to activation of ER stress and therefore induction of autophagy [101, 122]. Adipose-secreted hormone leptin enhances IL-1*β* secretion in beta cells, inhibits the secretion of IL-1R*α* (its natural antagonist), and therefore triggers the innate immune system in type 2 diabetes individuals [123, 124]. Moreover, IL-1*β* increases inflammation in beta cells by the IRE1*α* signaling pathway [125]. Elevated expression of TNF*α* and interferon gamma (IFN-*γ*) activates ER stress in human, rat, and mouse beta cells and nitric oxide (NO) production in rat [126]. Recent studies have demonstrated that salubrinal (a selective inhibitor of the PERK-eIF2*α* pathway) blocks TNF*α*, but not IL-1*β*, and thereby inhibits the NF*κ*Β signaling [127]. Manganese (III) meso-tetrakis (N-ethylpyridinium-2-yl) porphyrin (MnP) (manganese metalloporphyrin SOD mimetic) decreases iNOS, TNF*α*, and MCP-1 levels by blockade of NF*κ*Β in type 2 diabetes individuals [128]. Moreover, no pharmaceutical treatment but aerobic training decreased serum levels of IL-6, IFN-*γ*, TNF*α*, advanced oxidation protein products (AOPP), and thiobarbituric acid-reactive substances (TBARS) and in addition elevated levels of IL-10 and total thiol content (T-SH) in obese patients [129]. A better understanding of the ER stress response molecular mechanisms carries potential strategies to various metabolic disease treatments [130].

## 5. Future Perspective for Cellular Therapies

Stem cell transplantation is an excellent platform to metabolic syndrome-associated disease therapy, including obesity and type 2 diabetes [131, 132]. Recent preclinical and clinical studies have revealed that stem cell therapy had been applied successfully in diabetes mellitus individuals. Preclinical studies on an animal model have shown that MSC treatment exhibited a promising therapeutic effect on glycemic control through recovering islet function and improving insulin resistance. Approximately 100 registered phase I/II clinical trials among type 2 diabetes mellitus documented patients have been found with the clinical study registry [132, 133].

Because of their multipotential capacity, MSC is the most popular stem cell type used in diabetes treatment. A small amount of diabetic MSCs (6%) expresses both proinsulin and C-peptide; thus, MSCs possess the potential to differentiate into physiologically functional insulin-producing cells (IPCs) [30, 134, 135]. Moreover, MSCs promote the regeneration of endogenous pancreatic islet cells by secreting numerous cytokines and growth factors. The MSCs migrate to impaired islet cells and secrete paracrine factor, including insulin-like growth factor 1 (IGF-1), vascular endothelial growth factor (VEGF), angiopoietin-1, and platelet-derived growth factor BB (PDGF-BB) [136, 137]. MSCs have also immunoregulatory capacity because of the low intracellular expression of MHC class II [138]. Moreover, they suppress the proliferation of T lymphocytes and promote T-cell tolerance [139]. Additionally, MSCs inhibit the proliferation of B lymphocytes, thereby decreasing cytokine secretion, cytotoxicity of natural killer (NK) cell, and lymphocytes T, as well as B cell maturation and antibody production [140]. Additionally, MSCs promote autophagosome and autolysosome formation and thereby protect the islet cells [141].

MSCs have enzymatic and nonenzymatic mechanisms to inactivate ROS and to improve damages of genome and proteasome caused by reactive species that guarantee an efficiently managed OS [142, 143]. Studies have shown that rat MSCs, human BMSCs, and the immortalized cell line human MSC-telomerase reverse transcriptase (TERT) cultivated in the presence of ascorbate revealed expression of active thioredoxin reductases, catalase (CAT), glutathione peroxidases (GPXs), SOD1, and SOD2 [144, 145]. Calió et al. have reported that BMSC-based therapy reduces apoptosis rate and reactive oxygen species in the cell of rats with high blood pressure [146]. Furthermore, MSCs promote pancreatic islet against oxidative stress and hypoxia that cause cell destruction. Chandravanshi and colleagues have demonstrated that after 48 h of coculture with Wharton's jelly-derived MSCs, pancreatic islet cells exhibited increased viability, reduced apoptosis rate, and decreased levels of ROS, NO, and superoxide ions in comparison to the control group (without MSCs). In contrast to pharmaceutical antioxidant therapy, MSC can not only reduce oxidative stress (elimination of reactive oxygen species, free radicals) but also promote regeneration of previously damaged tissue [147, 148]. MSC transplantation was proven to be a very useful tool in the therapy of pathologies in which cell damage is linked to OS occurrence.

Unique properties of mesenchymal stem cells make them a suitable candidate for a number of metabolic disease therapies. However, some reports have demonstrated that MSC allogenic transplantation can lead to increased tumor transformation due to their immunosuppressive and multipotent features [132, 149]. Interestingly, most studies revealed that allogenic application is much more efficient in diabetes treatment than autologous transplantation [29, 150]. Moreover, aspects like the survival time of engrafted MSC *in vivo*, optimal dosing regimen, and the long-time effect of repeated dosing need further investigation [132]. In the future, mesenchymal stem cell therapy is expected to become a new level of therapeutic option for MetS-related diseases, but more controlled and advanced clinical trials are needed to optimize the application process.

## 6. Conclusions

Nowadays, metabolic syndrome and type 2 diabetes have become the leading causes of death among adults under the age of 60 and are highly associated with lifestyle. Excessive intake of sugar and fatty acids causes increased inflammation, ROS accumulation, and ER stress occurrence. A better understanding of the relationship between UPR, oxidative stress, insulin resistance, and inflammation will give new approach of the course of MetS. Application of ASC, as a promising tool for MetS and type 2 diabetes therapeutic intervention, is still hindered by technical and biological barriers.

## Figures and Tables

**Table 1:** Characterization of specific MSC surface biomarkers in non-MetS and MetS individuals.

	Specific surface markers	Characterization/effects	References
Non-MetS	(−) CD34, CD45	(−) hematopoetic stem cell-specific markers	[27, 28]
	(+) CD105, CD90, CD73, CD44, CD166	(+) mesenchymal stem cell-specific markers	

MetS	↓ CD90, CD105, CD73	↓ decreased clonogenic potential, increased PDT, decreased potential for chondrogenesis	[42–46]
	↑ CD44	↑ inflammatory response	[47–49]

MetS: metabolic syndrome; (+): presence; (−): lack; ↑: increase; ↓: decrease.

**Table 2:** Functions of adipokines and biomarkers in metabolic syndrome.

Adipokines/biomarkers	Function in metabolic syndrome	References
TNF*α*	↑ systemic insulin resistance via NF*κ*B/JNK signaling	[56, 58, 59]
	↓ insulin signal transduction	[68]

IL-6	↑ production of hepatic CRP	[69]
	↑ hyperglycemia, atherosclerosis	[63, 64]
	↓ insulin signal transduction	[70]
	↑ insulin resistance via NF*κ*B/JNK signaling	[58, 59]

PAI-1	↑ adipose tissue development	[65]
	↓ insulin signal transduction	[65]

MCP1	↑ insulin resistance via NF*κ*B/JNK signaling	[58, 59]

CRP	↑ atherosclerosis	[60]

TNF*α*: tumor necrosis factor-*α*; IL: interleukin; MCP-1: monocyte chemoattractant protein 1; PAI-1: plasminogen-activator inhibitor 1; CRP: C-reactive protein; ↑: increase; ↓: decrease.

## Abbreviations

MetS: Metabolic syndrome
ER: Endoplasmic reticulum
MSC: Mesenchymal stem cell
DPP-4: Dipeptyl-peptidase-4
SGLT2: Sodium glucose transporter-2
GLP-1: Glucagon-like peptide
SVF: Stromal vascular fraction
ASC: Adipose tissue-derived mesenchymal stem cell
BMSC: Bone marrow-derived mesenchymal stem cell
CFU-Fs: Fibroblast colony-forming units
PDT: Population doubling time
IL-1*β*: Interleukin 1*β*
MCP-1: Monocyte chemoattractant protein-1
TNF*α*: Tumor necrosis factor *α*
PAI-1: Plasminogen activator inhibitor-1
CRP: C-reactive protein
OS: Oxidative stress
SOD: Superoxide dismutase
ROS: Reactive oxygen species
GSH: Glutathione
Ca^2+^: Calcium cation
UPR: Unfolded protein response
IRE1*α*: Inositol-requiring enzyme 1*α*
PERK: Protein kinase RNA-like endoplasmic reticulum kinase
ATF6: Activating transcription factor 6
IR: Insulin resistance
BiP: Chain-binding protein
XBP-1: X-box binding protein 1
ERAD: ER-associated degradation
TRAF2: TNF*α* receptor-associated factor 2
JNK: Jun N-terminal kinase
ASK1: Apoptosis signal-regulating kinase-1
eIF2*α*: Eukaryotic translation initiation factor 2 alpha
ATF4: Activating transcription factor-4
CHOP: C/EBP*α*-homologous protein
Bcl-2: B cell lymphoma-2
S1P: Site 1 proteases
S2P: Site 2 proteases
IRS: Insulin receptor substrate-1
Bix: BIP inducer X
MAPK: P38 mitogen-activated protein kinase
RIPK1: Receptor-interacting serine/threonine-protein kinase 1
ISRIB: Integrated stress response inhibitor
MAMs: Mitochondria-associated ER membranes
PDI: Protein disulfide isomerase
AP1: Activator protein 1
NF*κ*Β: Nuclear factor *κ*Β
TLR4: Toll-like receptors 4
IFN-*γ*: Interferon gamma
NO: Nitric oxide
MnP: Manganese metalloporphyrin SOD mimetic
AOPP: Advanced oxidation protein products
TBARS: Thiobarbituric acid-reactive substances
T-SH: Total thiol
IPCs: Insulin-producing cells
IGF-1: Insulin-like growth factor 1
VEGF: Vascular endothelial growth factor
PDGF-BB: Platelet-derived growth factor BB
NK: Natural killer
TERT: Telomerase reverse transcriptase
CAT: Catalase
GPX: Glutathione peroxidase
EMS: Equine metabolic syndrome.
